# A thermo-sensitive peptide hydrogel loaded with paclitaxel and antimony nanosheets for synergistic photothermal-chemotherapeutic treatment of breast cancer

**DOI:** 10.3389/fphar.2025.1681276

**Published:** 2025-10-10

**Authors:** Bo Yan, Ling Gu, Hajra Zafar, Aiping Shi, Huanming Ge, Yu Jin, Nan Wang, Miao Wang, Binbin Liu, Yiqun Wu, Tianli Tian, Rongjie Ding

**Affiliations:** ^1^ Department of Pharmacy, Taixing People’s Hospital, Taixing, Jiangsu, China; ^2^ School of Pharmacy, Shanghai Jiao Tong University, Shanghai, China; ^3^ Nanjing Bond International College, Nanjing, Jiangsu, China; ^4^ School of Pharmacy, China Pharmaceutical University, Nanjing, Jiangsu, China; ^5^ Department of Pharmacy, Xuzhou Central Hospital, Xuzhou, Jiangsu, China; ^6^ Department of Oncology, Taixing People’s Hospital, Taixing, Jiangsu, China

**Keywords:** thermo-sensitive, hydrogel, PTX, photothermal therapy, cancer

## Abstract

**Introduction:**

Breast cancer is one of the most common cancers in the world. Paclitaxel (PTX), a potent chemotherapeutic agent, is widely used in cancer treatment but suffers from poor aqueous solubility and systemic toxicity.

**Methods:**

In this study, we developed a thermo-sensitive peptide, TSP-5, which forms a stable hydrogel at 37 °C and dissociates upon heating to 45 °C to facilitate drug release. The hydrogel was co-loaded with PTX and photothermal agent antimony nanosheets (AM) for targeted delivery to tumor sites. Under near-infrared (NIR) laser irradiation, AM rapidly elevated the local temperature due to its high photothermal conversion efficiency, triggering hydrogel dissolution and subsequent PTX release. Simultaneously, AM-mediated photothermal therapy (PTT) contributed to tumor cell ablation.

**Results:**

Both *in vitro* and *in vivo* studies demonstrated that the TSP-5 hydrogel significantly enhanced PTX solubility, reduced systemic toxicity, and improved anti-tumor efficacy.

**Discussion:**

The findings highlight its potential as a promising platform for localized drug delivery.

## 1 Introduction

Worldwide, cancer remains one of the most serious health challenges. Breast cancer is one of the three most common cancers (Harbeck and Gnant, 2017) and the most frequent malignancy in women (Harbeck and Gnant, 2017). This alarming figure highlights the urgency and complexity of the fight against breast cancer. Currently, radiotherapy (Kalbasi et al., 2017), chemotherapy (Chabner and Roberts, 2005) and operation (Wyld et al., 2015) are the most commonly used treatment. However, these traditional treatments often lack specificity and cause severe side effects (Li et al., 2019; Valle et al., 2017; Connor and Rose, 2018; Digklia and Wagner, 2016). Therefore, there is an urgent need to develop new treatment strategies to improve the effectiveness of treatment and reduce side effects.

Paclitaxel (PTX) is a widely used chemotherapeutic that promotes apoptosis by stabilizing microtubules and inhibiting cell division. However, its clinical application is limited by poor solubility, nonspecific distribution, and adverse effects such as myelosuppression and neuropathy. Various nanocarriers have been developed to improve PTX delivery while reducing its side effects (Chen et al., 2018; Zhang et al., 2019; Ge et al., 2018), including nanoparticles, liposomes, and polymers, yet challenges remain regarding biocompatibility and controlled release (Schiff and Horwitz, 1980; Whitaker and Placzek, 2019; Jelinek et al., 2015; Tudor et al., 2000; Yang et al., 2020; Marupudi et al., 2007).

Injectable self-assembling peptide hydrogels have emerged as attractive drug delivery systems due to their high biocompatibility and tunable release profiles (Raza et al., 2019; Liu et al., 2019; Shen et al., 2017; Xu et al., 2016). These hydrogels can form a three-dimensional network structure *in vivo*, enabling sustained and localized drug delivery. Recent advances have incorporated stimuli-responsive mechanisms (e.g., temperature or pH sensitivity) for spatiotemporal control over drug release to improve therapeutic efficacy and reducing side effects (Zhang et al., 2016; Lan et al., 2015; Zhao et al., 2018; Mei et al., 2019).

Two-dimensional nanomaterials such as antimony nanosheets (AM) exhibit excellent photothermal conversion efficiency and have shown great potential in biomedical applications (Tao et al., 2017; Tao et al., 2018). Photothermal therapy (PTT) utilizes light-absorbing nanomaterials to generate localized heat under NIR irradiation, selectively killing cancer cells. Combining PTT with chemotherapy offers a synergistic approach to enhance treatment efficacy while reducing drug doses and side effects.

Despite the promising progress in stimuli-responsive drug delivery systems, a significant challenge remains in achieving precise spatiotemporal control over drug release to maximize therapeutic efficacy while minimizing systemic toxicity. Conventional thermosensitive hydrogels often rely on passive drug diffusion or bulk degradation for release, which is poorly synchronized with the dynamic therapeutic demand at the tumor site.

**SCHEME 1:**
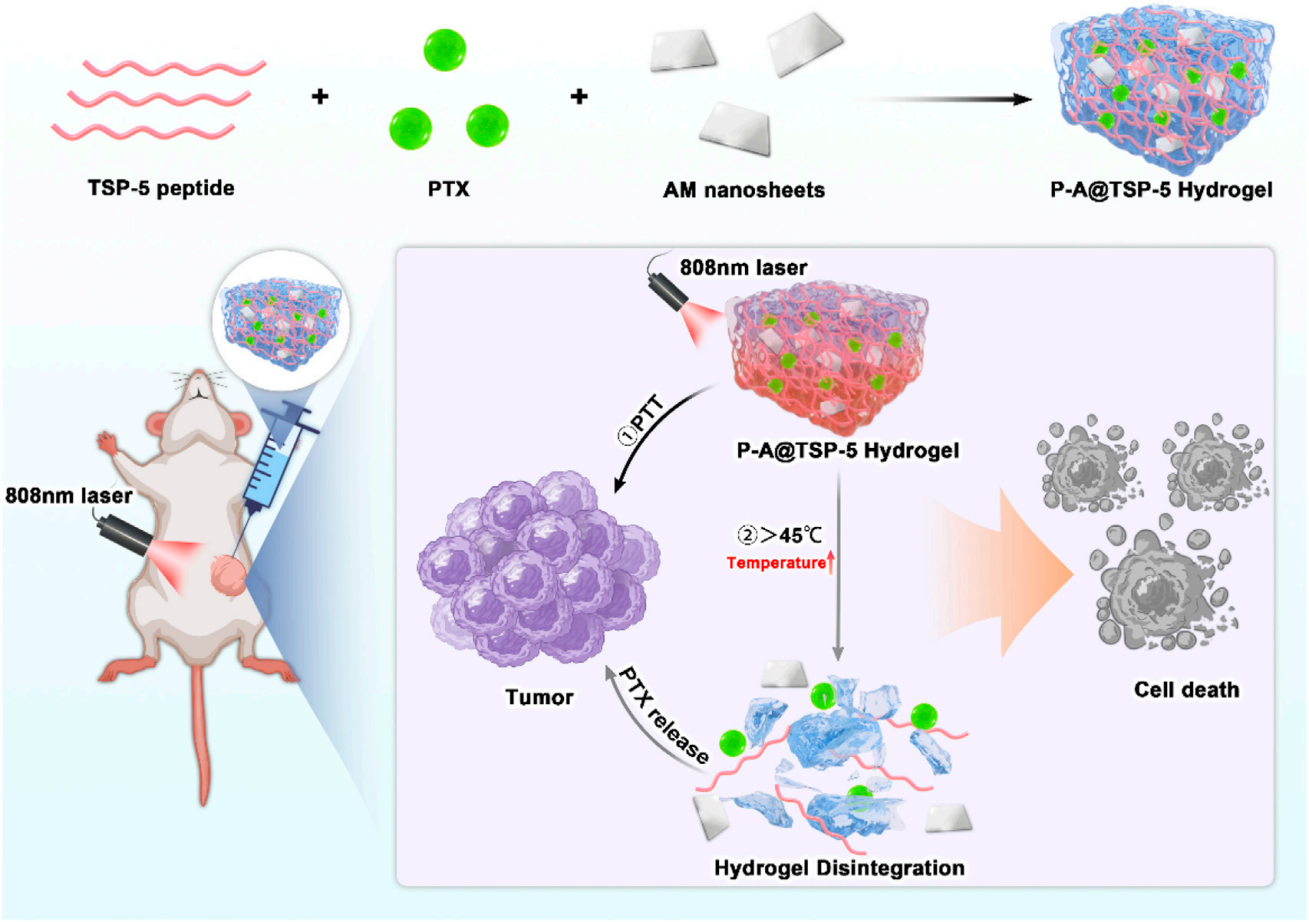
TSP-5 Hydrogel could effectively kill tumor cells through both PTT and enhanced PTX release under 808 nm near-infrared laser, realizing the combination of PTT and chemotherapy.

In this study, we address this gap by engineering a co-delivery platform where the thermos-sensitive peptide hydrogel acts not merely as a passive reservoir, but as an active gatekeeper. The phase transition behavior of thermo-sensitive TSP-5 hydrogel is meticulously aligned with the photothermal profile of the antinomy nanosheets (AM), which ensures that the hydrogel maintains structural integrity at physiological temperature (37 °C) for sustained retention, yet undergoes rapid and complete dissolution upon mild photothermal heating (to ∼45 °C), triggering a burst release of the chemotherapeutic agent PTX, thus achieving a combination of photothermal therapy and chemotherapy (Scheme 1). This synergistic orchestration of material properties to create a highly responsive ‘on-off’ switch for drug release offers a promising platform for combinatorial cancer therapy.

## 2 Materials and methods

### 2.1 Materials

Fmoc Protective Amino Acid, Wang Resin, N′, N’ -diisopropyl carbodiimide (DIC), 1-hydroxybenzotriazole (HOBt), acetic anhydride, purchased from Shanghai Gill Biochemical Company. Dichloromethane (DCM) was purchased from Nanjing Chemical Reagent Company. N′, N-dimethylformamide (DMF) was purchased from Nanjing Haibang Trading Company. 1, 2-ethyldimercaptan (EDT) was purchased from Shanghai Bangcheng Chemical Company. Triethylsilane (TIS) was purchased from Suzhou Jinghua Chemical Company. Piperidine is purchased from Shanghai Lingfeng Chemical Reagent Company. N, n-isopropyl carbodiimide (TFA) was purchased from Nanjing Aikang Company, antimony and paclitaxel (PTX) were purchased from Shanghai Aladdin Reagent Company. DMEM medium and CCK-8 kit were purchased from KeyGEN BioTECH.

4T1 cell line were purchased from Wuhan Pricella Biotechnology Co., Ltd. and cultured in a cell culture incubator (37 °C, 5% CO_2_). The culture medium used in this research was DMEM medium (10% v/v FBS, 1% v/v penicillin streptomycin).

BABL/c mice (female, 6 weeks old) were purchased from Jiangsu Qinglongshan Biotechnology Co., Ltd.

### 2.2 Preparation and characterization of antimony nanosheets

Antimony nanosheets (AM) were synthesized via ultrasonic exfoliation. Antimony powder suspended in anhydrous ethanol was sonicated for 30 h in an ice bath. The supernatant was collected after centrifugation (3,000 rpm, 10 min, 4 °C), followed by further centrifugation (12,000 rpm, 30 min, 4 °C) to pellet the nanosheets. The precipitate was resuspended in deionized water and lyophilized for subsequent use.

The microscopic morphology of AM was observed by transmission electron microscopy (HT-7700, Hitachi), and size distribution was determined using a Zetasizer Nano ZS (Malvern).

Photothermal properties of AM: Different concentrations (0, 0.2, 0.4, 0.6, 0.8, 1 mg/mL) of AM suspension were prepared, and irradiated with 1 W/cm^2^ 808 NIR light for 10 min was adopted, the power density was set to 1.0W/cm^2^, and the continuous laser irradiation was performed for 10 min.

Photothermal stability of AM: 0.6 mg/mL AM suspension was placed in a 2 mL EP tube and irradiated with 808 nm near-infrared light with a power of 1.0 W/cm2 at room temperature for 5 min. Subsequently, the irradiation stopped, and the sample was allowed to cool naturally to room temperature. A total of 5 cycles were performed.

The photothermal experiments above were conducted with single-mode fiber coupled laser series (LWIRPD-5F, Beijing Lae-Zhiwei Photoelectric Technology Co., LTD.) for irradiation and infrared thermal imager (HT-H8, Dongwan Xintai Instrument Company) for temperature recording. The temperature change curves were drawn according to the statistics.

### 2.3 Synthesis and purification of thermo-sensitive polypeptides

The peptide TSP-5 (Ac-DDIII-VPGVG-K-NH_2_) was synthesized via Fmoc/tBu solid-phase peptide synthesis on Wang resin. After chain assembly, cleavage was performed using a mixture of TFA/EDT/TIS/water (95:2:2:1). The crude peptide was purified by RP-HPLC (Shimadzu LC-8A), and molecular weight was confirmed by ESI-MS.

### 2.4 Preparation and characterization of TSP-5 hydrogels

The gelation behavior of TSP-5 was evaluated at various concentrations, temperatures, and AM concentrations. Gel formation was assessed via vial inversion.

Concentration study: At room temperature (25 °C), aqueous solutions of TSP-5 was prepared at concentrations of 15 mg/mL, 20 mg/mL, 25 mg/mL and 30 mg/mL, respectively, and adjusted to pH 7.4 with NaOH (0.5 M) and HCl (0.1 M). Gel formation was assessed via vial inversion.

Temperature study: Aqueous solutions of TSP-5 (20 mg/mL) were prepared and then placed in water bath at 30 °C, 37 °C, 40 °C, 45 °C, 50 °C and 55 °C respectively to observe the gelatinization.

AM concentration study: Aqueous solutions of TSP-5 (20 mg/mL) was prepared at 25 °C and AM suspension of different concentrations (0.2, 0.4, 0.6, 0.8 and 1.0 mg/mL) was added. The sample was then placed in a water bath at 37 °C and 45 °C respectively to observe the gelatinization.

Appearance of the hydrogel: Gel sample was prepared with TSP-5 (20 mg/mL), PTX (2 mg/mL), and AM (0.6 mg/mL) in vial at 37 °C, and the state of the gel under the inclined state was recorded. In addition, an identical sample was prepared in parallel, placed in a water bath at 45 °C for 30 min, and the state of the gel under the inclined state was also recorded.

Circular dichroism (CD): Two identical TSP-5 samples were heated in a water bath at 37 °C and 45 °C for 30 min, respectively, and then diluted to 0.5 mg/mL as the sample for CD detection. The circular dichroism (J-810, Nippon Spectroscopy Co., Ltd.) is set to detect in the wavelength range of 190–260 nm; the spectral band width is 1 nm; the wavelength response time is 1 s; the sweep speed is 50 nm/min.

Rheological test: The energy storage modulus and loss modulus of the polypeptide hydrogel were determined using a rotating rheometer (HAAKE 600, Thermo Fisher Technologies, United States). A circular lamina with a diameter of 25 mm was fixed. After 20 min of preheating at 25 °C, drops of the two polypeptide hydrogel samples were added to the test bench to ensure consistent gel thickness and no bubbles, and the upper lamina was slowly lowered. The rheological test was performed after the hydrogel stood for 30 min. The fixed frequency is 6.28 rad/s and the strain scanning range is 0.1%–100% for dynamic strain sweep. Set the frequency to 0.1–100 rad/s and the shear force to 1% for dynamic frequency sweep. Set the frequency to 6.28 rad/s, shear force to 0.1%–1%, scan 0–120 s, increase the shear force to 50%, scan for 120 s, and then set the shear force back to 0.1%–1%, scan for 120 s, repeat the process to complete the cycle sweep.

### 2.5 Drug release

Gel sample (100 μL; 20 mg/mL TSP-5, 2 mg/mL PTX, 0.6 mg/mL AM) was prepared at 37 °C in a 1.5 mL EP tube. PBS (pH 7.4) was used to carefully wash the surface of the sample, and the cleaning solution was collected. The content of PTX not encapsulated was then determined by HPLC, and the encapsulation rate was calculated according to the formula: *Encapsulation rate (%) = (PTX*_*total*_ *–PTX*_*in cleaning solution*_ *)/PTX*_*total*_ *× 100%*.

1 mL PBS (pH 7.4) was added as release medium, and the EP tube was placed in a thermostatic oscillator (120 rpm, 37 °C). All the release solution were carefully collected at different time points (0.5, 2, 4, 8, 12, 24, 48, 72, 96, 120, 144 and168 h), and the same volume of fresh release media was added. For the laser group, samples were irradiated (808 nm, 1 W/cm^2^, 5 min) before each sampling. PTX release was quantified by HPLC.

### 2.6 Biocompatibility *in vitro*

100 μL A@TSP-5 was prepared and 1 mL culture medium was added as release medium in EP tubes. EP tubes were placed in a thermostatic oscillator at 120 rpm and 37 °C for 0 (that is, fresh culture medium), 24, 48, 72, 96, 120, 144 and 168 h respectively, and the release solution was collected with the same method of laser group in section 2.5.

4T1 cells were seeded in a 96-well plate (1 × 10^5^ cells per well) and cultured for 24 h. Later, the culture medium was removed and 100 μL release solution was added to each well. In addition, culture medium was added to wells without cells as blank group. 24 h later, each well was washed with PBS, and 100 μL fresh culture medium (10% v/v CCK-8 reagent) was added. After 2 h of further cultivation in the incubator, the OD value of each well at the wavelength of 450 nm was measured by the enzyme-labeled instrument EL-x800, and the relative cell viability of each group was calculated according to the formula: *Relative cell viability (%) = (OD*_*target*_ *-OD*_*blank*_ *)/(OD*_*control*_ *-OD*_*blank*_ *) × 100%*.

### 2.7 Anti-tumor efficacy *in vitro*

4T1 cells were seeded in a 96-well plate (1 × 10^5^ cells per well), cultured for 24 h, and divided into Free PTX group, P-A@TSP-5 group and P-A@TSP-5 + L group. The concentration of PTX in gel was the same as free PTX. 4T1 cells were then co-incubated with free PTX (0.1, 1, 10, 20, 50 μg/mL) or the 48-h release solution of gel for 24 h, and the relative cell viability of each group was measured by a CCK-8 kit.

A@TSP-5 was prepared according with section 2.6 to specifically evaluate the anti-tumor effect mediated solely by AM-induced PTT. 4T1 cells were seeded in a 96-well plate (1 × 10^5^ cells per well), cultured for 24 h, and divided into Control group, A@TSP-5 group, L/A@TSP-5 group, A@TSP-5 + L group, and L/A@TSP-5 + L group. A@TSP-5 group: release solution was collected from hydrogel without laser irradiation, and then co-cultured with cells; L/A@TSP-5 group: release solution was collected from hydrogel without laser irradiation, and then co-cultured with cells; “+ L” indicates that irradiation (1 W/cm^2^ 808 nm laser, 5 min) was conducted during co-culture. The relative cell viability of each group was measured by a CCK-8 kit after 24 h of further incubation.

### 2.8 Anti-tumor efficacy *in vivo*

BABL/c mice (female, 6 weeks old) were subcutaneously injected of 4T1 cells (2 × 10^6^ cells) to establish 4T1 tumor model. When the tumor volume reached about ∼100 mm^3^, the mice were randomly divided into 3 groups (6 mice per group, 18 mice in total). Tumor-bearing mice were treated with saline, P-A@TSP-5, or P-A@TSP-5 + L, respectively. The injection volume was 100 μL/20 g. Mice in P-A@TSP-5 + L were irradiated by laser (808 nm, 1 W/cm^2^) for 5 min at the injection site every 2 days. Tumor volume of mice in each group were measured by vernier caliper every 2 days, and calculated with the following equation: *Tumor volume = length × width* ^*2*^ *× 0.5*. In addition, tumor inhibition rate was calculated with the equation: *Inhibition rate (%) = (1-V*_*sample*_ */V*_*control*_ *) × 100%*. The body weight of mice was recorded every 2 days. The mice were euthanized when the tumor volume of any mouse reached 1,000 mm^3^, and the tumor masses were dissected for TUNEL staining.

### 2.9 Biocompatibility *in vivo*

Healthy mice were divided into two groups (3 mice per group, 6 mice in total), and injected with saline or A@TSP-5 hydrogel (100 μL/20 g). 14 days later, mice were euthanized and main organs (heart, liver, spleen, lung, kidney) were collected for H&E staining. In addition, routine blood tests were performed.

### 2.10 Statistical analysis

Data were presented as the mean ± SD of a minimum of three samples (n ≥ 3). The number of repeated experiments or the number of experimental samples is indicated in the figure captions. All of the statistical analysis was carried out with GraphPad Prism 10 software. For comparisons of three or more groups, One-way ANOVA with Tukey’s multiple comparison test was performed. For comparisons of two groups, a two-tailed unpaired t-test was performed. The significances were represented by the marks: ns, no significance, p > 0.05; *, p < 0.05; **, p < 0.01; ****, p < 0.0001.

## 3 Results and discussion

### 3.1 Preparation and characterization of antimony nanosheets

**FIGURE 1:**
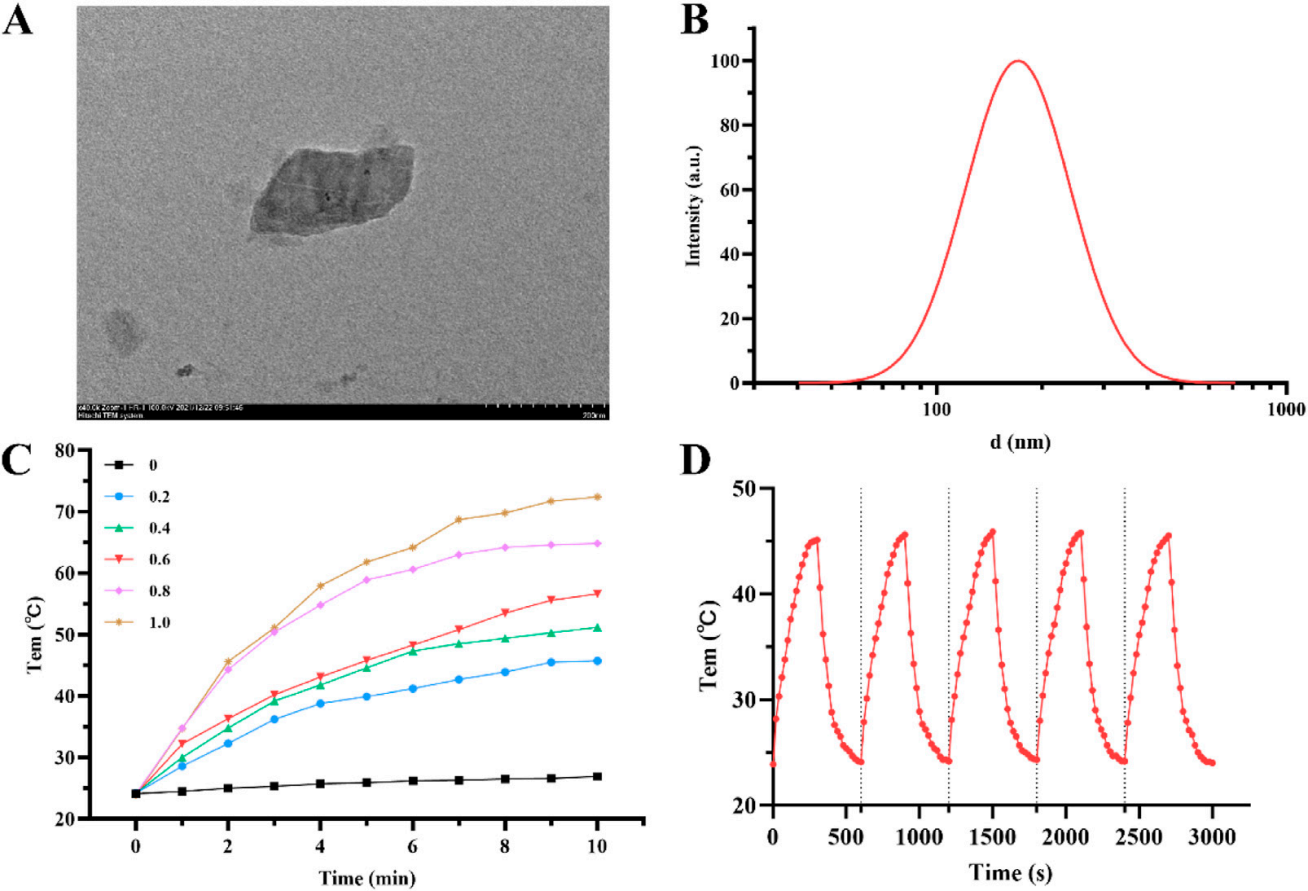
**(A)** TEM image of AM. **(B)** Particle size distribution of AM. **(C)** Temperature changing curves of 0, 0.2, 0.4, 0.6, 0.8 and 1 mg/mL AM dispersion under irradiation (808 nm, 1.0 W/cm^2^) for 10 min. **(D)** Temperature changing curves of AM dispersion over five consecutive laser (808 nm, 1.0 W/cm^2^) “on” and “off” cycles.

Antimony nanosheets (AM) were prepared by ultrasonic exfoliation (Wu et al., 2024). TEM revealed that AM exhibited a sheet-like morphology with an average size of ∼200 nm (Figure 1A). DLS indicated a hydrodynamic diameter of ∼170 nm (Figure 1B).

A single-mode fiber-coupled series laser with power of 1.0 W/cm^2^ and wavelength of 808 nm was used to irradiate AM dispersions of different concentrations for 10 min, and infrared thermal imager was used to measure the temperature of these samples at different time points, so as to study the photothermal properties of AM (Dibaba et al., 2019). Under NIR irradiation, AM dispersions showed time-dependent and concentration-dependent temperature increases, indicating its good photothermal properties (Figure 1C). It was worth noting that when the concentration of AM dispersion reaches 0.6 mg/mL, the temperature can reach ∼45 °C after 5 min of laser irradiation, meeting the temperature requirement of killing tumor cells. In addition, AM exhibited excellent photothermal stability, with which more flexible photothermal therapy could be achieved through multiple irradiations in clinical treatment: in the five heating-cooling cycles, the temperature of AM suspension reached 45.1 °C, 45.6 °C, 45.9 °C, 45.8 °C and 45.5 °C, respectively (Figure 1D).

### 3.2 Design, synthesis and characterization of thermo-sensitive peptides

**FIGURE 2:**
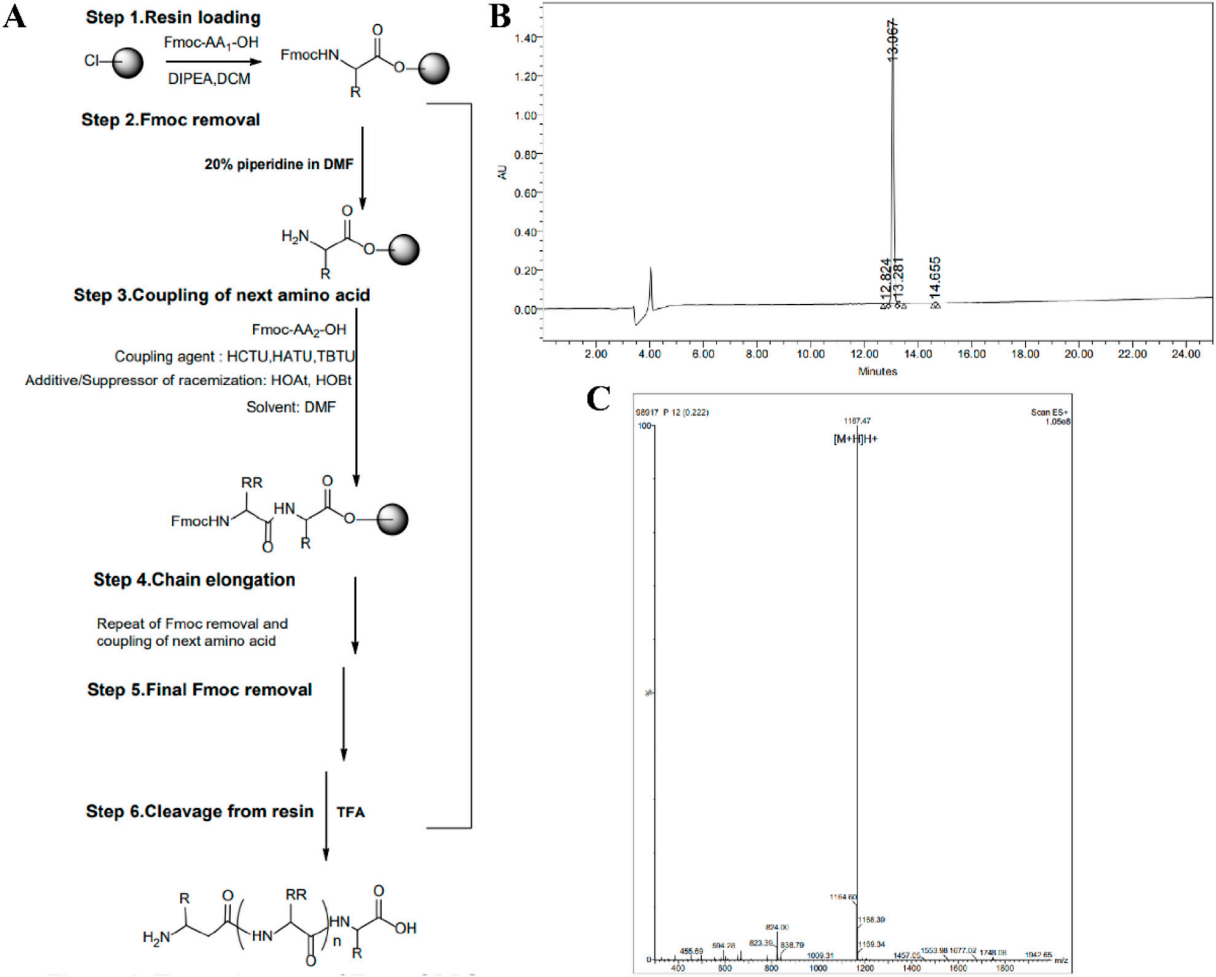
**(A)** Synthetic of polypeptide using solid phase synthesis method. **(B)** UPLC spectrum of TSP-5 peptides. **(C)** Mass spectrum of TSP-5 peptides.

We used the highly hydrophobic valine (Val, V) as the structural unit of Elastin-like polypeptides (ELPs), valine-proline-glycine-x-glycine (VPGXG), where the guest amino acid X is any amino acid except proline, and a short peptide with sequence VPGVG is obtained. Moreover, since the thermo-sensitive nature of ELPs is not lost due to its covalent binding to other proteins or peptides (Amiram et al., 2013; Christensen et al., 2013; Varanko et al., 2020), we introduced the DDIII short peptide sequence (Clarke et al., 2018), which can improve the ability of gel formation, and the water-soluble lysine (Lys, K) residue, which can improve the short peptide segment (Trevino et al., 2007), Finally, a polypeptide with the sequence Ac-DDIII-VPGVG-K-NH_2_ (TSP-5) was obtained. The peptide was synthesized by solid-phase synthesis method (Figure 2A), purified by RP-HPLC (Figure 2B), and its molecular weight was confirmed by LC-MS (Figure 2C).

### 3.3 Preparation and characterization of TSP-5 hydrogels

The gelation behavior of TSP-5 was evaluated under various conditions. We first studied the selection of peptide concentrations. As shown in Supplementary Table S1, no hydrogel formation was observed at pH 7.4 across concentrations of 15–30 mg/mL at 25 °C. A concentration of 20 mg/mL was selected for subsequent experiments based on solubility.

The gelation temperature was then investigated. As shown in Supplementary Table S2, under neutral conditions (pH 7.4), TSP-5 aqueous solution (20 mg/mL) was able to form a stable hydrogel at physiological temperature (37 °C), which gradually disintegrates with increasing temperature and becomes a completely viscous liquid at 45 °C. This is because TSP-5 polypeptide sequence contains more Isoleucine (Ile), which has a high tendency to form β folding. As a kind of ELPs, VPGVG can undergo reversible phase transition with temperature change and has LCST (low critical transition temperature) behavior (Liu et al., 2010). In addition, as a self-assembled hydrogel sequence, DDIII (Clarke et al., 2018) combines with VPGVG to play a role, which not only greatly improves the gelation rate and stability, but also enables reversible transformation of nanofiber secondary structure under the stimulation of different temperatures, thus better forming thermo-sensitive polypeptide hydrogels.

Next, the effect of the concentration of photothermal agent on the formation of gel was investigated. As shown in Supplementary Table S3, a stable gel could be formed at pH 7.4 °C and 25 °C. This may be due to the hydrophobic interaction between AM under the stimulation of temperature, and the binding of the repeating unit VPGVG and DDIII, resulting in the transformation of the nanofiber secondary structure from random curling to β-folding, which makes them intertwine and interact with each other. A stable hydrogel AM was formed. In addition, the time to gelation decreased with the increase of AM suspension concentration. When the concentration of AM suspension was 0.6 mg/mL, the gelation time was 30 min. On this basis, the gelation time did not change much as the concentration of AM increased. This may be because the gel has a certain loading limit, and when the loading amount is within the limit, it can reflect the better gelation performance. Therefore, 0.6 mg/mL was chosen as the concentration of AM for subsequent experiments.

In summary, we finally selected 20 mg/mL TSP aqueous solution loaded with photothermic agent AM (0.6 mg/mL) and chemotherapeutic drug PTX (2 mg/mL, as determined by previous studies (Sang et al., 2021)) to prepare the final hydrogel (P-A@TSP-5).

**FIGURE 3:**
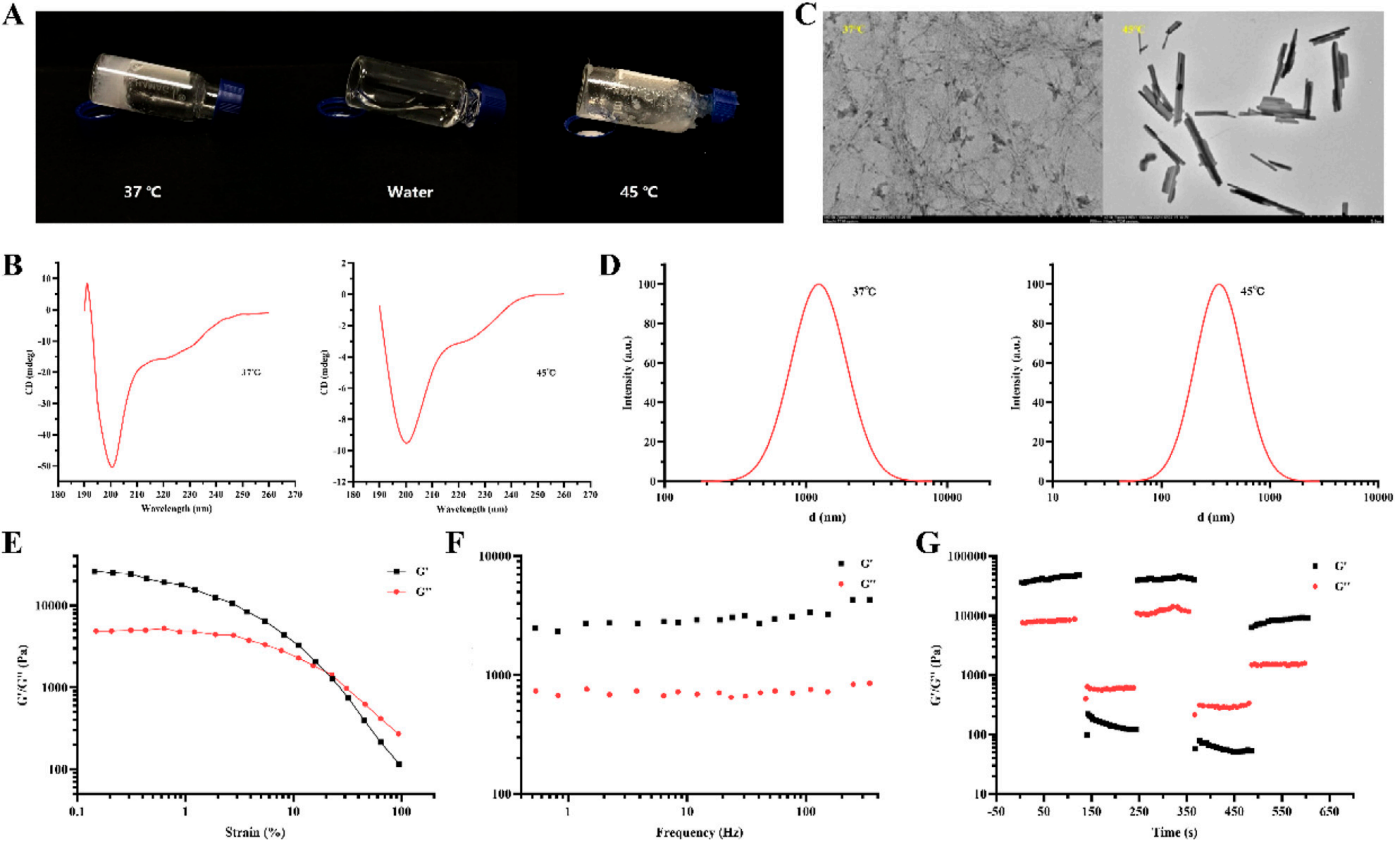
Appearance **(A)**, Circular dichroism **(B)**, TEM **(C)** and particle size distribution **(D)** of TSP-5 hydrogel under different temperature conditions. Dynamic strain sweep **(E)**, dynamic frequency sweep **(F)** and circle sweep **(G)** of TSP-5 hydrogel.

The thermos-sensitivity of the hydrogel was visually confirmed: the appearance of the hydrogel in the inclined state is shown in Figure 3A. With aqueous solution (middle of Figure 3A) as the control, at 37 °C (left of Figure 3A), the peptide solution did not scatter or flow in the inclined state, forming a relatively stable hydrogel. At 45 °C (right of Figure 3A), the peptide solution showed a viscous and fluid state, at which time the hydrogel had completely disintegrated.

In addition, to further characterize the temperature response of the peptide self-assembly behavior from a microscopic perspective, the secondary structure of the TSP-5 hydrogel was observed using circular dichroism (CD) (Figure 3B). At 37 °C, a positive peak appeared at 192 nm and a negative peak at 201 nm. The characteristics of the peak pattern were consistent with the β-fold structure, indicating that the secondary structure of TSP-5 was mainly β-fold structure, so stable hydrogel could be formed at 37 °C. At 45 °C, the circular two-dimensional chromatogram showed an obvious negative peak at 198 nm, which was consistent with the peak pattern characteristic of the random curl structure, indicating that the β-fold structure was destroyed at 45 °C and transformed into an unstable random curl structure, so the gel changed into a sol state. TEM imaging (Figure 3C) showed an interconnected nanofibrillar network at 37 °C, which disaggregated upon heating (45 °C). DLS indicated a hydrodynamic size reduction from ∼1,300 nm (37 °C) to ∼350 nm (45 °C) upon temperature increase (Figure 3D).

Rheological tests were performed on TSP-5 (Figures 3E–G). The results of dynamic strain sweep (Figure 3E) show that when the stress reaches the gel point (Lee et al., 2008), the gel changes from elastic dominance (elastic modulus G’ > viscous modulus G″) to viscous dominance (G’ < G″), and at this time the hydrogel changes from non-flowable to flowable. These results indicate that TSP-5 polypeptide hydrogels are solid-like materials with good mechanical properties. The dynamic frequency sweep results are shown in Figure 3F. In the whole scanning frequency range, G′ is greater than G″, and the variation of both is not large. The results showed that the gel state was a viscoelastic solid with strong rigidity, and the hydrogel formed by polypeptide had good stability. During drug delivery, the gel would not collapse and cause drug leakage or sudden release, and it was a stable carrier material. The cycle sweep results are shown in Figure 3G. When 50% shear force is applied to the gel, G″ is greater than G′, which indicates that the sample is in a liquid state and has fluidity at this time. When the shear force is reduced to 1%, G′ is greater than G″, and the sample returns to a solid-like state. This indicated that the polypeptide hydrogel could be used for injection.

### 3.4 Drug release

**FIGURE 4:**
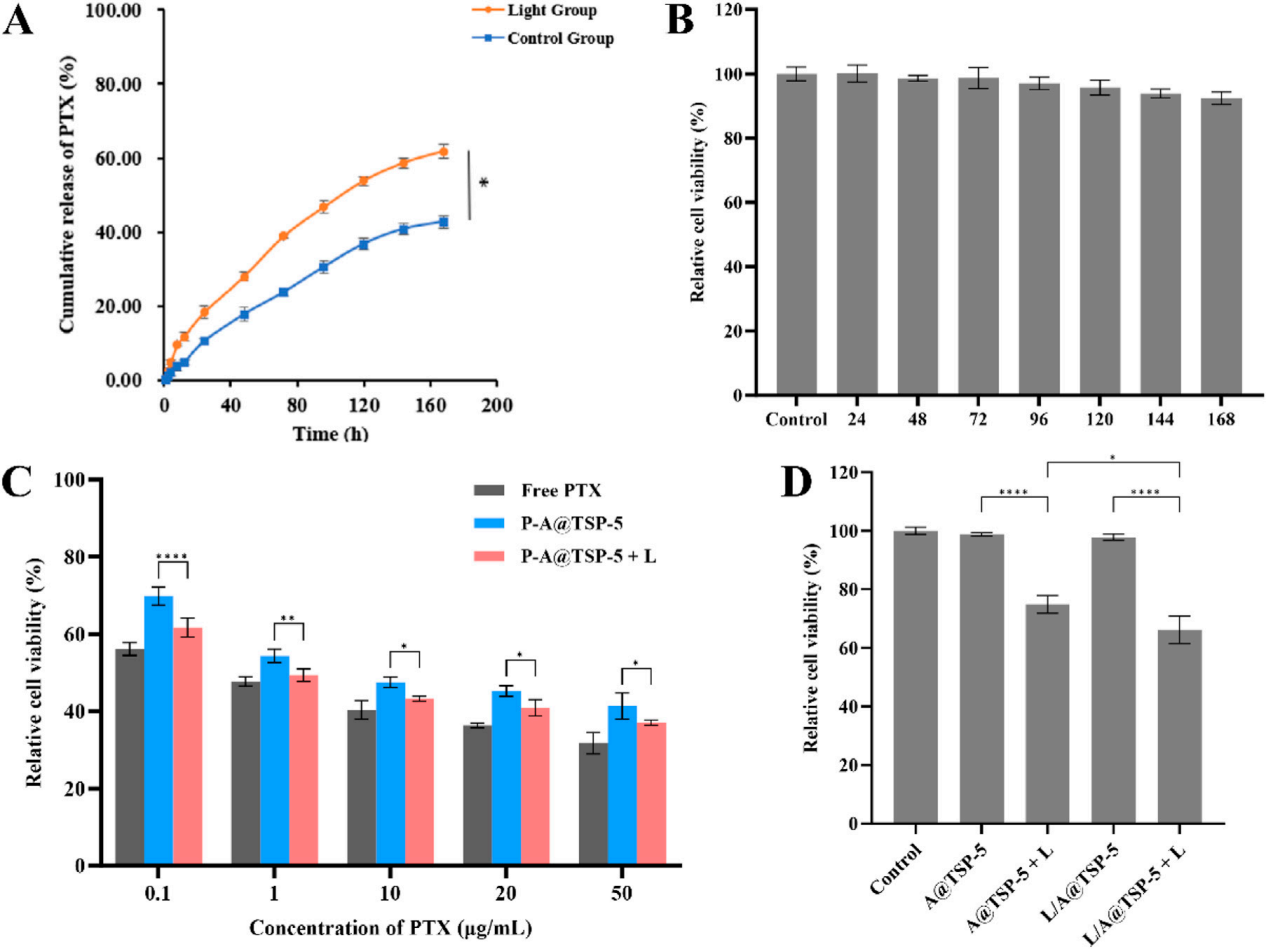
**(A)** Cumulative release of PTX from TSP-5 hydrogel without (Control group) or with (Laser group) light (n = 3). **(B)** Relative cell viability of 4T1 cells treated with A@TSP-5 (n = 3). **(C)** Relative cell viability of 4T1 cells treated with Free PTX, P-A@TSP-5, and P-A@TSP-5 + L (n = 3). **(D)** Relative cell viability of 4T1 cells treated with A@TSP-5, A@TSP-5 + L, L/A@TSP-5 and L/A@TSP-5 + L (n = 3). Data are presented as mean ± SD. One-way ANOVA with Tukey’s multiple comparison test: *, p < 0.05; **, p < 0.01; ****, p < 0.0001.

The cumulative release of PTX from the hydrogel reached 42.81% without laser and 61.83% with laser irradiation over 7 days (Figure 4A), confirming the on-demand release capability triggered by photothermal heating.

### 3.5 Biocompatibility *in vitro*

Before the *in vivo* experiment, the biocompatibility test was performed to ensure that the gel carrier is safe and non-toxic. TSP-5 hydrogel with AM encapsulated (A@TSP-5) was prepared for the evaluation of the biocompatibility. As shown in Figure 4B, when co-incubated with the release solution of A@TSP-5 at different time, the relative cell viability of 4T1 cells remained above 90%, indicating that A@TSP-5 itself had good biocompatibility and almost no carrier toxicity *in vitro*.

### 3.6 Anti-tumor efficacy *in vitro*

The cytotoxicity was investigated to verify the anti-tumor efficacy *in vitro*. Relative cell viability of 4T1 cells treated with different concentrations of free PTX and P-A@TSP-5 (with or without laser) were shown in Figure 4C. It could be seen that P-A@TSP-5 showed cytotoxicity in a PTX-dose-dependent manner. Consistent with the result of drug release mentioned above, the release of PTX could be enhanced when the temperature increased over 45 °C under the laser irradiation, so that P-A@TSP-5 + L group exhibited greater cytotoxicity than P-A@TSP-5 group. In addition, it was worth noting that both P-A@TSP-5 group and P-A@TSP-5 + L group showed slighter cytotoxicity than free PTX when the concentration of PTX administered was the same, indicating the slow-drug-release capability of the gel which could allow administration of higher dosage of drug.

On the other hand, AM led to apoptosis of tumor cells by triggering PTT (Figure 4D). AM exhibited no significant cytotoxicity against 4T1 cells without irradiation, demonstrating the biocompatibility of AM. Once irradiated with laser, AM triggered PTT to kill tumor cells. The relative cell viability of L/A@TSP-5 + L group was lower than that of A@TSP-5 + L group because there were more AM released from the gel in the release solution.

### 3.7 Anti-tumor efficacy *in vivo*

**FIGURE 5:**
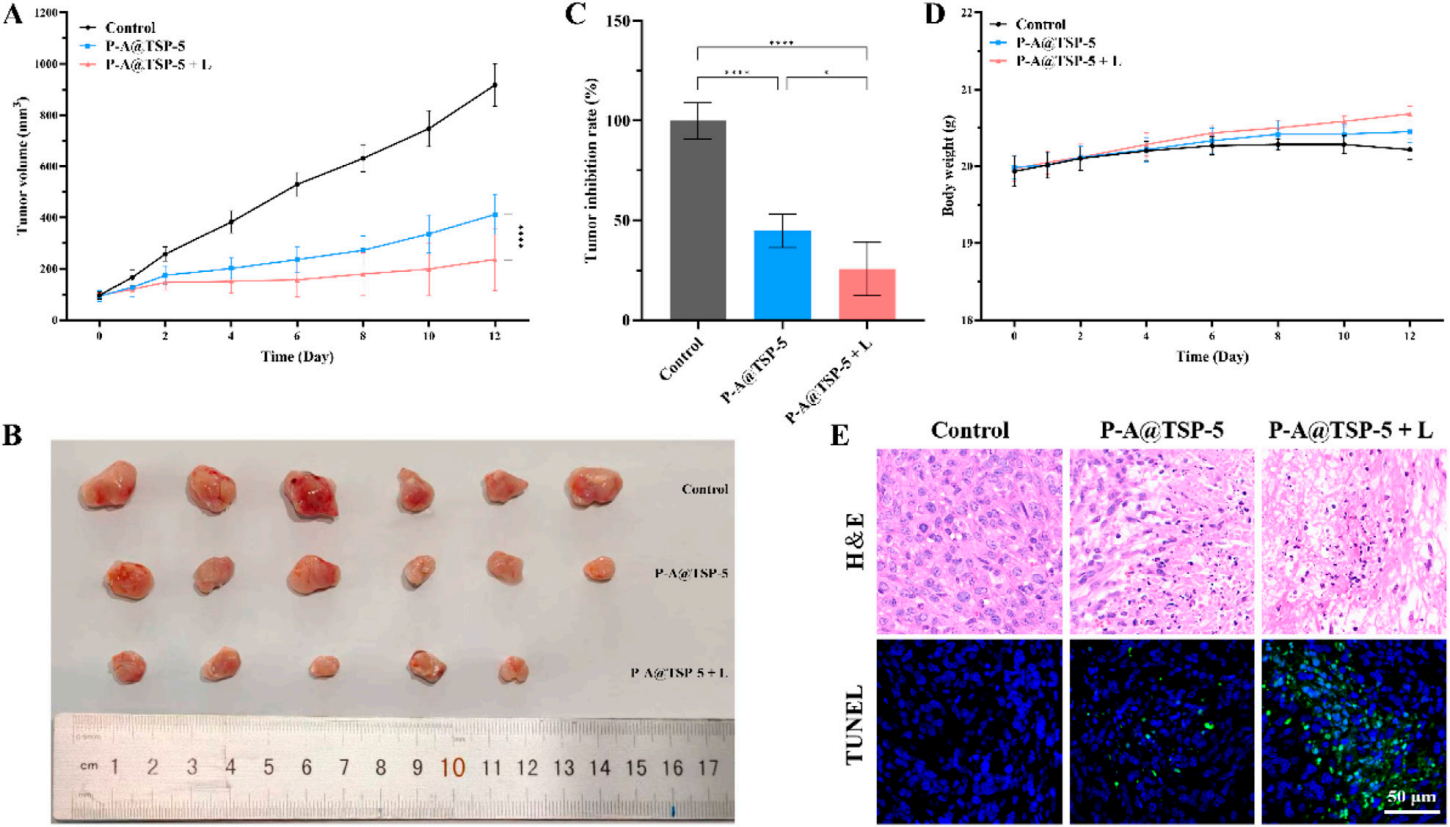
**(A)** Tumor volume curves for tumor-bearing mice under different treatments (n = 6). **(B)** Image of tumor tissues dissected after treatment. **(C)** Tumor inhibition rate of P-A@TSP-5 and P-A@TSP-5 + L (n = 6). **(D)** Body weight of mice within the treatment period (n = 6). **(E)** H&E and TUNEL staining images of tumor tissues dissected after treatment. Data are presented as mean ± SD. One-way ANOVA with Tukey’s multiple comparison test: *, p < 0.05; ****, p < 0.0001.

To further study the anti-tumor efficacy of P-A@TSP-5 hydrogel, BABL/c mice were subcutaneously injected of 4T1 cells and treated with saline (Control group), P-A@TSP-5, or P-A@TSP-5 + L, respectively. As shown in Figures 5A–C, compared with the control group, both P-A@TSP-5 group and P-A@TSP-5 + L group were able to effectively inhibit the growth of tumor. It is worth noting that treatment efficacy of P-A@TSP-5 + L group was better than that of P-A@TSP-5 group, and complete tumor suppression was observed on 1 mouse of P-A@TSP-5 + L group. This could be attributed to the enhanced release of PTX resulting from the temperature increase caused by AM (Supplementary Figure S1), and PTT triggered by AM may kill tumor cells to some degree as mentioned in section 3.6. In other words, the combination of chemotherapy and photothermal therapy effectively inhibits tumor growth.

The body weight of mice within the treatment period was recorded (Figure 5D). The body weight of the control group mice first showed a slight increase, and then showed a decreasing trend. We believe this was due to the slow growth rate of the tumor initially, which did not lead to weight loss in the mice; as the tumor volume grew faster, the body weight of mice trended to decrease. On the contrary, the body weight of mice of P-A@TSP-5 group did not show a decreasing trend after the increase in the first few days, which could be attributed to the anti-tumor efficacy of PTX. The body weight of mice of P-A@TSP-5 + L group even kept increasing because of the enhanced tumor inhibition caused by the enhanced release of PTX.

In addition, we further carried out H&E and TUNEL staining of the tumor tissue (Figure 5E) in order to evaluate the anti-tumor efficacy. H&E staining of tumor tissues of P-A@TSP-5 + L group showed enhanced degree of tissue necrosis compared with P-A@TSP-5 group, which was due to the enhanced release of PTX. TUNEL staining showed that P-A@TSP-5 + L group presented the largest percentage of apoptotic cells, consistent with the results of H&E staining.

### 3.8 Biocompatibility *in vivo*

**FIGURE 6:**
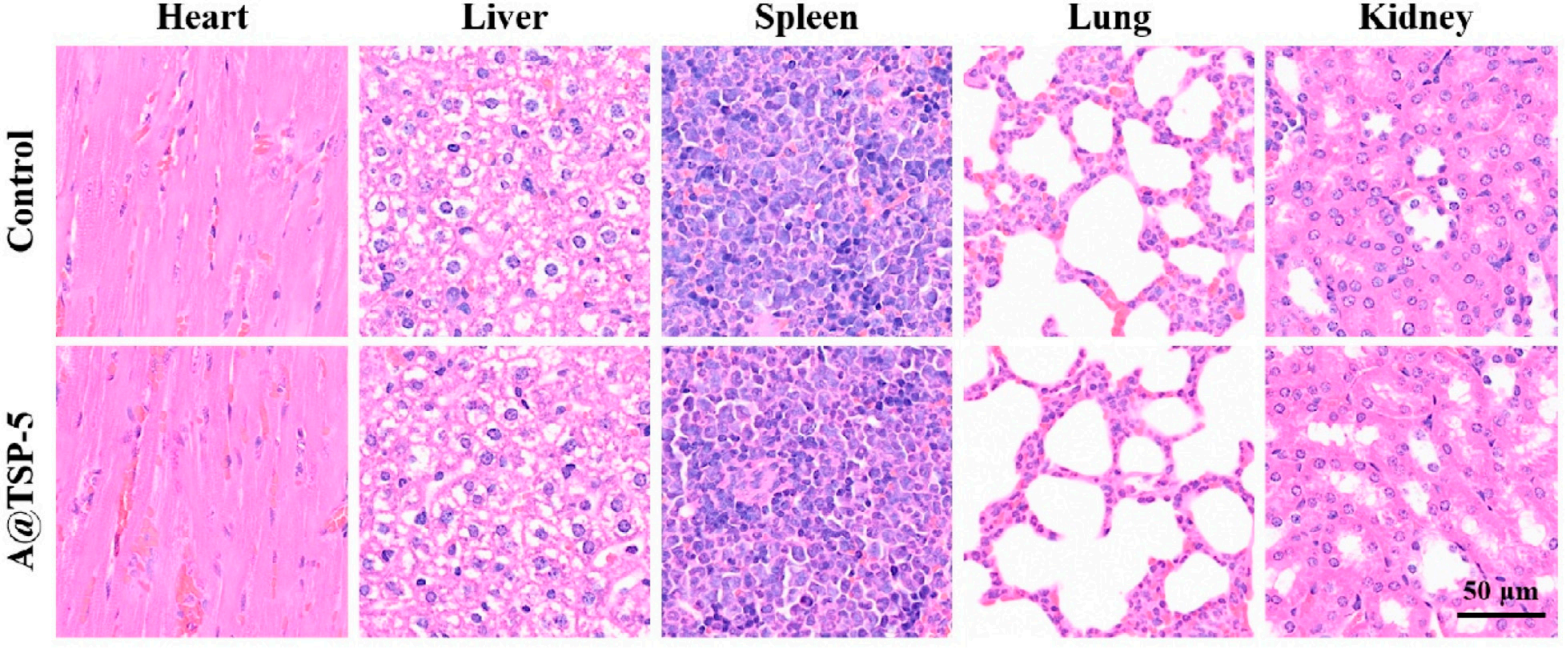
H&E staining of main organs dissected from healthy mice injected with saline (Control group) and A@TSP-5 hydrogel after 14 days.

**FIGURE 7:**
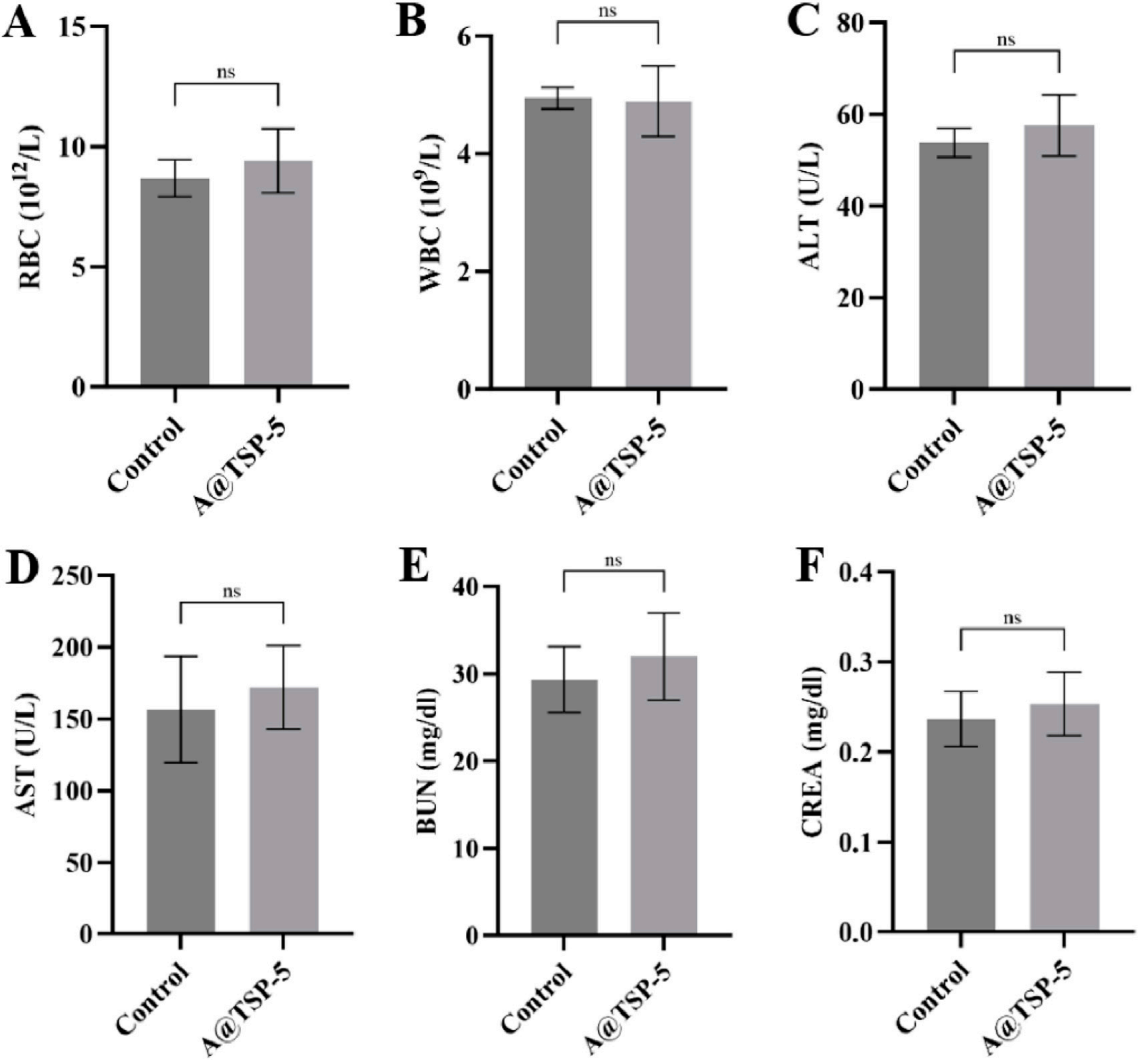
Hemogram and biochemical indexes of healthy mice injected with saline (Control group) and A@TSP-5 hydrogel after 14 days (n = 3). **(A) **RBC; **(B)**WBC; **(C)** ALT; **(D)** AST; **(E)** BUN; **(F)** CREA. Data are presented as mean ± SD. Two-tailed unpaired t-test: ns, no significance, p > 0.05.

H&E staining (Figure 6) of main organs dissected from healthy mice injected with saline (Control group) and A@TSP-5 hydrogel for 14 days showed no obvious pathological changes (inflammation or tissue necrosis); routine blood tests (Figure 7) showed no significant difference between the two groups, indicating that both AM and TSP-5 hydrogel had supreme biocompatibility *in vivo*, which was consistent with the results of *in vitro* experiment.

## 4 Conclusion and discussion

In summary, we have successfully engineered an injectable thermosensitive hydrogel-based platform (TSP-5) for the co-delivery of antimony nanosheets (AM) and the chemotherapeutic drug paclitaxel (PTX). This system effectively addresses critical challenges associated with PTX, including its low solubility and high systemic toxicity, by providing localized and on-demand drug release. Comprehensive rheological characterization confirmed that the TSP-5 hydrogel possesses excellent mechanical strength, stability, and shear-thinning properties, ensuring its suitability as an injectable depot.

The novelty of our platform transcends the mere combination of existing components. Its core innovation lies in the synergistic design where the gel-sol transition temperature of the peptide is strategically set to be triggered by the photothermal effect of the embedded AM nanosheets under mild NIR irradiation. This ingenious coupling enables precise spatiotemporal control over drug release, allowing for a potent burst of PTX precisely when and where it is needed. It is crucial to highlight that the AM nanosheets serve a dual therapeutic function: firstly, as a photothermal agent to directly ablate tumor cells through mild PTT, and secondly, as a nanoscale heater to disrupt the hydrogel matrix and actuate the on-demand release of PTX. This level of multi-functional control is unattainable with conventional sustained-release systems.

Consequently, *in vivo* studies demonstrated significant suppression of 4T1 tumor growth, attributable to the potent synergistic interplay between two distinct therapies: the direct cell-killing effect of AM-mediated PTT and the triggered chemotherapy from the released PTX. Importantly, the spatial confinement offered by the hydrogel depot ensures that both the hyperthermia and the high-concentration drug release are concentrated within the tumor microenvironment, thereby markedly mitigating off-target effects and enhancing treatment safety.

We believe that this multifaceted strategy—combining localized depot formation, photothermal-triggered precise drug release, and the synergistic interplay of chemo-photothermal therapy—provides a robust, controllable, and highly promising blueprint for enhancing the precision and efficacy of solid tumor treatment.

## Data Availability

The original contributions presented in the study are included in the article/Supplementary Material, further inquiries can be directed to the corresponding authors.
