# Supplementary material for: A thermo-sensitive peptide hydrogel loaded with paclitaxel and antimony nanosheets for synergistic photothermal-chemotherapeutic treatment of breast cancer
Source: Front Pharmacol. 2025 Oct 10;16:1681276. doi: 10.3389/fphar.2025.1681276 (PMC12549564; doi:10.3389/fphar.2025.1681276)
Supplement: Supplementary file 1 [file Supplementaryfile1.docx]

**Supplementary materials**

**Tab. S1** Solubility of TSP-5 at different concentrations

| Peptide | Concentration of peptide (mg/mL) | | | |
| --- | --- | --- | --- | --- |
|  | 15 | 20 | 25 | 30 |
| TSP-5 | L | L | SS | IS |

L: liquid; IS: insoluble; SS: slightly soluble

**Tab. S2** Gelation of TSP-5 under different temperature

| Peptide | Temperature (℃) | | | | | |
| --- | --- | --- | --- | --- | --- | --- |
|  | 30 | 37 | 40 | 45 | 50 | 55 |
| TSP-5 | L | VL:10 min  SG: 32 min | SG | UG: 30 min  VL: 1 min | VL | VL |

L: liquid; VL: viscous liquid; SG: stable gel; UG: unstable gel

**Tab. S3** Gelation time of TSP-5 with different concentration of AM loaded at different temperatures

| Temperature (℃) | Concentration of AM（mg/mL） | | | |
| --- | --- | --- | --- | --- |
|  | 0.2 | 0.4 | 0.6 | 0.8 |
| 37 | VL:15 min  SG: 40 min | VL: 14min  SG: 33 min | VL:10 min  SG: 30 min | VL:9 min  SG: 30 min |
| 45 | UG:39 min  VL: 3 min | UG:33 min  VL: 4 min | UG:30 min  VL: 2 min | UG:30 min  VL: 2 min |

VL: viscous liquid; SG: stable gel; UG: unstable gel


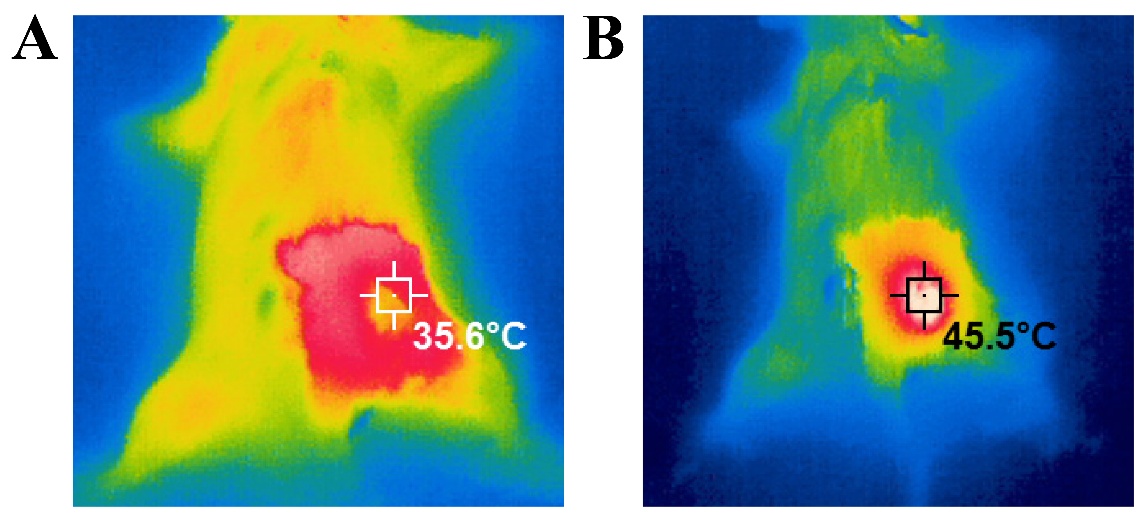


Fig. S1 Representative infrared thermal images of BABL/c mice before (A) and after (B) NIR (808 nm, 1 W/cm^2^) irradiation for 5 min
